# Gut Microbiome and Risk of Lethal Prostate Cancer: Beyond the Boundaries

**DOI:** 10.3390/cancers15235681

**Published:** 2023-12-01

**Authors:** Pranav Prakash, Shiv Verma, Sanjay Gupta

**Affiliations:** 1 College of Arts and Sciences, Case Western Reserve University, Cleveland, OH 44106, USA; pxp468@case.edu; 2 Department of Urology, School of Medicine, Case Western Reserve University, Cleveland, OH 44106, USA; sxv304@case.edu; 3 The Urology Institute, University Hospitals Cleveland Medical Center, Cleveland, OH 44106, USA; 4 Department of Pharmacology, Case Western Reserve University, Cleveland, OH 44106, USA; 5 Department of Pathology, Case Western Reserve University, Cleveland, OH 44106, USA; 6 Department of Nutrition, Case Western Reserve University, Cleveland, OH 44106, USA; 7 Division of General Medical Sciences, Case Comprehensive Cancer Center, Cleveland, OH 44106, USA

## 1. Introduction

The gut microbiome is critical in balancing human health and in influencing the risk of several chronic diseases, including cancer [1]. It is also involved in metabolism, nerve transmission, blood circulation, and the functioning of the immune system, and its association with several human diseases such as arthritis, cardiovascular disease, and diabetes is well established [2]. A growing body of evidence indicates that the gut microbiome is associated with limited human malignancies [3]. Dysbiosis or disruption of gut microbial colonies allows microbes to spread to various organs through interorgan network via systemic circulation. Establishment of these new microbial colonies often generates an immunosuppressive microenvironment and chronic inflammation, increasing the risk of cancer development. Individuals with colorectal, prostate, and pancreatic cancer exhibit dysbiosis in the oral–gut axis, which affects disease pathogenesis. In prostate cancer patients, microorganisms such as *Alistipes* and *Lachnospira*—known producers of short-chain fatty acids—were present in higher amounts in the gut, suggesting a possible relationship between the metabolite(s) and risk of prostate cancer progression [4]. A combination of factors resulting from an imbalance in the gut microenvironment may also play a significant role in the increased risk of lethal prostate cancer. A study by Reichard et al. [5] highlighted the dependency of metabolic pathways on the risk of lethal prostate cancer conducted on cohorts from the PLCO screening trial. The authors utilized the serum samples from the PLCO trial to demonstrate a relationship between the levels of certain metabolites and the increased risk of lethal prostate cancer.

## 2. Background

The PLCO screening trial was a large randomized controlled study that recruited 150,000 men and women aged 55 to 74 in the United States. The study aimed to determine the effects of screening modalities on Prostate, Lung, Colon, and Ovarian Cancer. Screening Centers were established in ten different states and patients were enrolled from 1993 to 2001. Supplemental data and biospecimens, including blood samples, buccal cells, and tumor tissue cores and microarrays, were collected from patients during follow-up until 2011. The PLCO database has been used as an epidemiological resource, allowing researchers to conduct scientific studies [6]. Serum samples from the PLCO screening trial have also allowed for investigations into the relationship between metabolites and cancer susceptibility.

The authors utilized PLCO screening trial as a source for baseline collection of serum samples from 173 lethal prostate cancer and 519 control individuals in a nested case–control study. Controls had no prostate cancer present before the screening trial or during the study. Race and age were also taken into consideration in matching cases of death due to lethal prostate cancer to controls at a 1:3 ratio. The metabolites, choline, carnitine, betaine, γ-butyrobetaine, crotonobetaine, trimethyl N-oxide (TMAO), phenylacetylglutamine (PAGln), hippuric acid, and p-cresol sulfate levels were quantified from healthy individuals and patients with lethal prostate cancer. The serum samples were analyzed via liquid and mass chromatography and monitored through electrospray ionization to create general calibration curves. Using a linear regression model, median baseline levels of the metabolites were compared between lethal prostate cancer and control cases. Higher levels of choline/betaine, carnitine, PAGln, and p-cresol sulfate in men with lethal prostate cancer were found to be statistically significant. Higher levels of betaine, however, did not exhibit a trend in association with lethal prostate cancer, whereas carnitine showed inconsistent association in the conditional multivariable logistic regression model. PAGln demonstrated a dose-dependent relationship, while p-cresol sulfate, hippuric acid, γ-butyrobetaine, and crotonobetaine were inconsistent in association [5].

## 3. Discussion

The interplay between the gut microbiota and the incidence and progression of human cancers are limited [7]. Thus far, few reports have established an association between gut microbiome and prostate cancer. A study conducted by Liss et al. (2018) was the first to report the relationship between human prostate cancer and the gut microbiome [8]. The results revealed that folate and arginine metabolism pathways were enriched in patients with prostate cancer. Simultaneously, oral microbiota, as the second largest microbial habitat in the body, has been linked with the development of numerous diseases [3,9]. The translocation of oral microbiota due to dysfunction in the oral barrier allows microbes to invade and cause inflammation in foreign areas [10,11]. Oral microbial displacement has been associated with colorectal, pancreatic, and prostate cancer [11,12,13]. In these cancers, oral microbes produce chronic inflammation, increasing the risk of cancer development. For example, Porphyromonas gingivalis, a bacteria associated with periodontitis, was found to promote the growth of infected cancerous cells [12]. Through dysbiosis, oral bacteria colonize in the prostate, leading to an increased risk of prostate cancer [14]. While the relationship between certain analytes and an increased risk of lethal prostate cancer has been demonstrated by Reichard et al. [5], the source of analytes and their link with the gut microbiome has not been firmly established. The source of the metabolites, whether it is generated through the endogenous pathways or the microbiome-dependent pathway, is lacking. The study failed to establish a definite association between higher levels of choline, betaine, and PAGln with the microbiota or microbial pathways. A discussion of these factors would allow a link between the levels of analytes to dysbiosis of the gut microbiome. The production of metabolites could potentially be an interplay of endogenous pathways and alteration in the microbiome affecting prostate cancer lethality. Increased presence of certain bacteria could induce greater production of prostate cancer-associated metabolites in malignant cells. Without an encompassing discussion of the analytic pathway, an association between the found metabolites and the gut microbiome cannot be established. Prostate cancer-associated metabolites found by the authors could be validated by in vitro or in vivo models in association with gut microbiome-dependent pathways in a controlled study [5].

## 4. Conclusions

Additional studies are needed to understand the microbial source and potential role of gut microbiome-dependent pathway(s) in increasing the risk of lethal prostate cancer. Further in-depth studies into the gut–prostate axis are required to reach a definite conclusion.
